# The evolutionary ecology of nests: a cross-taxon approach

**DOI:** 10.1098/rstb.2022.0136

**Published:** 2023-08-28

**Authors:** Mark C. Mainwaring, Mary Caswell Stoddard, Iain Barber, D. Charles Deeming, Mark E. Hauber

**Affiliations:** ^1^ School of Natural Sciences, Bangor University, Bangor LL57 2DG, UK; ^2^ Department of Ecology and Evolutionary Biology, Princeton University, 106A Guyot Hall Princeton University, Princeton, NJ 08544-2016, USA; ^3^ Department of Life Sciences, Aberystwyth University, Aberystwyth, Ceredigion SY23 3DA, UK; ^4^ Joseph Banks Laboratories, School of Life and Environmental Sciences, University of Lincoln, Lincoln LN6 7DL, UK; ^5^ Department of Evolution, Ecology, and Behavior, School of Integrative Biology, University of Illinois at Urbana-Champaign, Urbana, IL 61801, USA

**Keywords:** architecture, behaviour, evolution, extended phenotypes, nest construction, reproduction

## Abstract

Nests, including the enormous structures housing colonies of eusocial insects and the elaborately built nests of some fishes, have long fascinated scientists, yet our understanding of the evolutionary ecology of nests has lagged behind our understanding of subsequent reproductive stages. There has, however, been a burgeoning amount of interest in nests over the past decade, and this special issue on ‘The evolutionary ecology of nests: a cross-taxon approach' outlines our understanding of the form and function of nests in diverse animal lineages. Papers in ‘The function of nests: mechanisms and adaptive benefits' theme examine the various functions of nests, while papers in ‘The evolution of nest characteristics' theme examine the evolution of nesting behaviours. Meanwhile, papers in the ‘Large communal nests in harsh environments' theme examine how the enormous structures constructed by eusocial insects and social birds enable them to inhabit harsh arid environments, whereas papers in the ‘Nests in the Anthropocene' theme examine how adaptive shifts in nest architecture allow animals to adapt to breed in the age of accelerating global human impacts. Finally, the synthesis outlines how the mixture of ideas and approaches from researchers studying different taxa will advance our understanding of this exciting field of research.

This article is part of the theme issue ‘The evolutionary ecology of nests: a cross-taxon approach’.

## 1. Introduction

Nests are built by a range of vertebrate and invertebrate taxa—including fishes, reptiles, amphibians, birds, mammals and insects—to house vulnerable eggs and offspring [1,2]. Nest structures typically hold the eggs of oviparous species and the offspring of both oviparous and viviparous species and, therefore, play a key role in achieving reproductive success [1,3–5]. For example, Medina *et al*. [5] showed that interspecific variation in the design of birds' nests is an important determinant of the evolutionary persistence, and thus success, of species. Nevertheless, our understanding of the evolutionary ecology of nests and nest building has lagged behind our understanding of the later stages of reproduction, such as incubation behaviours and offspring provisioning, despite the fact that nests—including the large burrows of mammals and the enormous structures that house colonies of eusocial insects—have long held a fascination for scientists [1].

The past decade or so, however, has seen an increasing focus on the nests built by a range of nest-constructing taxa, including fishes, amphibians, birds, non-avian reptiles and mammals [1,2,6–11]. The recent increase in attention is due, in no small part, to the assembly of nearly exhaustive datasets of all the extant avian species [12], which when combined with the simultaneous resolution of their phylogenies, is allowing comparative studies to examine the evolution of nest characteristics in a phylogenetically robust manner [5,13,14].

Meanwhile, an array of modern descriptive and analytical techniques, including computational approaches, enables the quantification of the shape of nests from digital images and other scans [15–17]. Studies of nests stored in museums also provide new possibilities to examine temporal and spatial variation in nest characteristics in a level of detail not previously possible [18–20]. This range of novel and updated approaches has vastly advanced our understanding of the evolutionary ecology of nests by enabling researchers to perform studies that were impossible just a few years ago.

To date, many studies examining nests have focused on single species, and topics have been treated in an isolated manner, meaning that the full extent of the inter-disciplinary nature of the field has not been synthesized [2]. This special issue aims to address some of these limitations by assembling papers that examine the evolutionary ecology of nests and nest building behaviour across taxonomic boundaries, while using a variety of focal systems to better understand how variation in nest characteristics evolved [14,21]. The mixture of ideas and approaches from researchers studying different taxa will synergistically combine to advance our understanding of the topic [1].

The issue also showcases how some of the novel techniques outlined above are transforming our understanding of the evolutionary ecology of nests [18,22,23]. Further, the papers within the issue will also have implications for concepts such as nest-inspired architecture—a phenomenon exemplified by the Beijing National Stadium, also known as the ‘Bird's Nest', which hosted the 2008 Beijing Olympic Games—which has recently gained momentum [24].

The papers in this special issue are grouped into four conceptual themes. The first theme is entitled ‘The function of nests: mechanisms and adaptive benefits' and examines how selective pressures from predators and reproductive partners influence nest architecture in taxa including fishes, amphibians and mammals. The second theme is entitled ‘The evolution of nest characteristics' and includes papers that use data from thousands of species of birds and insects to examine the evolution of nest architecture in a phylogenetically controlled manner to better understand the factors that drive the evolution of diverse nest architecture. The third theme, entitled ‘Large communal nests in harsh environments' examines the function of the incredibly large communal nests of social insects and birds in harsh arid environments in the Southern Hemisphere. Finally, the fourth theme entitled ‘Nests in the Anthropocene' contains papers that examine how shifts in nest building behaviours may enable animals such as fish, turtles and other reptiles to adapt to changing environmental conditions and explore the extent to which different bird species incorporate anthropogenic materials into their nests.

## 2. The function of nests: mechanisms and adaptive benefits

Nests are structures that primarily serve to provide locations in which eggs and offspring can develop. The predation of eggs or offspring causes reproductive failure and so natural selection exerts considerable selective pressures on the design of nests [25,26]. Yet, nests also serve as extended phenotypic signals of the builder's quality [27] and are thus subject to selective pressures as a result of sexual selection.

While the majority of research examining these issues has focused on birds (e.g. [27]), studies of fishes have also proved informative (reviewed in [8]). Empirical studies of three-spined sticklebacks (*Gasterosteus aculeatus*) show that their nests play an important role in courtship behaviours [28,29]. However, while natural selection favours small and inconspicuous nests, sexual selection favours larger and conspicuous nests and the resolution of this evolutionary trade-off is examined here by Svensson & Kvarnemo [30].

Svensson & Kvarnemo [30] show that the design of ray-finned fish nests is driven by both natural and sexual selection. Their nests range from bowls, burrows and ridges, to nests made of algae or bubbles and the design of their nests plays an important role in sexual selection by protecting individuals against sperm competition or nest usurpations. However, the relative contributions of natural and sexual selection to nest structure remains unclear. Consequently, Svensson & Kvarnemo [30] highlight several species that readily build nests and breed in aquariums and are likely to further elucidate the relative contributions of natural and sexual selection in determining the design of nests of ray-finned fish [30].

Meanwhile, nest building is widespread in mammals [31–33]. However, Deeming [34] reviews those studies that have examined nest building in mammals and reports that many mammals use nests for maternity roles, but in many species there are a wide range of other roles, including resting, environmental protection and as hibernacula, which are not seen in birds. Very few of those studies provided insights into the determinants of variation in the array of nest building materials used, particularly when compared to the better-studied birds. Consequently, more studies are needed to increase our understanding of the function of mammal nests [34].

The materials used to build nests are known to vary at the interspecific [35] and intraspecific [36] levels in wild birds. The cause of such variation is difficult to establish in wild birds, but studies of captive birds show that older individuals build neater woven nests than younger conspecifics [37] and that they exhibit evidence of learning by adaptively selecting nest materials in relation to their colour and physical characteristics [38,39]. Lehtonen *et al*. [40] review the prevalence of learning in nest-building across a range of taxa and conclude that learning is ubiquitous in nest-building animals, yet they also outline many areas of research where further research is most warranted [40].

Environmental factors, too, can affect nesting behaviours and thereby influence nest microclimates. This is important because the temperature that offspring experience within nests can influence their phenotypes. de Jong *et al*. [41] examine whether developmental temperatures influence among-individual variation and repeatability in both thermal plasticity and trait means by incubating delicate skink (*Lampropholis delicata*) eggs at three different temperatures and quantifying locomotor performance and resting metabolic rate as juveniles and as adults. Nest temperature has a lasting effect on growth and locomotor performance, with cool and hot incubation temperatures resulting in faster growth and larger maximum size, and hot incubation temperatures reducing locomotor performance at all timepoints [41], thus demonstrating that nest microclimates have long-lasting effects on individuals.

Finally, the nest building behaviours of anurans are relatively poorly understood, particularly when compared to other taxa, although various studies have examined nest-building behaviours in frogs [42,43]. Here, Fischer [44] outlines our current understanding of the nest-building behaviour of anurans, while also focusing on topical issues, such as why poison frogs surround their eggs with egg-jelly while they are incubated within nests. She concludes that the egg-jelly has three main functions that benefit reproduction in nest-building frogs: first, it contains toxins and thus provides a chemical defence against predators and microbes; second, it helps to glue tadpoles to the back of the frogs; and third, it helps terrestrially breeding species to maintain aquatic developmental environments for the tadpoles [44].

## 3. The evolution of nest characteristics

The evolution of nests is perhaps best understood in birds [14,25,45,46] and we also have a good, and ever-increasing, understanding of the nests of the non-avian ancestors of birds [47]. Incomplete skeletons of the sauropodomorph *Massospondylus*, which is a genus of prosauropod dinosaurs from the early Jurassic Period, showed that *Massospondylus* deposited a single layer of tightly packed eggs below ground [48].

Hogan & Varricchio [49] outline that while most dinosaurs likely buried their single layer of highly porous eggs underground, pennaraptoran theropods only partially buried their far less porous eggs. The shift to laying even partially exposed eggs represents a major transition in the evolution of nesting and Hogan & Varricchio [49] propose that nest guarding by endothermic archosaurs may have resulted in an indirect form of contact incubation, which selected for shallower clutch burial and an increasing benefit from temperature provided by the parent. The continued selection pressure may well have led to the transition of fully subaerial eggs seen in extant birds.

The evolution of nest architecture and nest sites in birds and their non-avian ancestors is reviewed by Mainwaring *et al*. [50] and they highlight a trend of nests being located in increasingly exposed locations. There has also been a pattern towards nests becoming less substantial yet increasingly elaborate, particularly in passerine birds, which has been accompanied by parents laying fewer eggs and providing an increasing amount of time per offspring over evolutionary timescales [50].

Several of the papers in this special issue further advance our understanding of the evolution of birds' nests. Ocampo *et al*. [51] examine the evolution of nest architecture of tyrant flycatchers and their allies and they show that the Tyrannida ancestor likely built a cup nest in a closed habitat, although domed nests evolved at least 15 times quite independently within the group. Cup nesting and domed nesting species both diversified to inhabit open and semi-open habitats, indicating that there were no coevolutionary relationships between habitat and nest type. Further, Ocampo *et al*. [51] did not find a link between nest type and a range of life history and environmental traits, suggesting that no one factor determines the evolution of nest architecture in the Tyrannida [51].

In addition to data compiled from online data sources, there has also been a recent shift to using nests held in museum collections [18,20,52]. Perez *et al*. [52] use more than 700 nests from 55 passerine species held in museums to examine the link between nest design and behavioural flexibility, which is likely to be an important determinant of survival for species in a changing world. Intraspecific variation in nest traits was greater in those species with domed nests than those with open cup nests, and in species in which nests were built by a single parent, while also exhibiting a high phylogenetic signal. However, nest building flexibility is not linked to behavioural flexibility [52].

Meanwhile, Sheard *et al*. [53] examine evolutionary relationships between nest characteristics and birds' bills, which is interesting because it is the first study to link the morphological measurements of birds' bills and the material/s they use to build their nests, while also taking foraging behaviour and phylogeny into account. Using data from nearly 6000 species worldwide, it is shown that beak morphology and also access to materials and species diet predict the use of nest material. Despite the relationships being heavily influenced by phylogenetic signal and sampling biases, so that the strength of the relationships is driven by phylogenetic inertia, there is nonetheless a link between the morphology of birds' beaks and the material composition of their nests [53].

The evolution of nests is best described in birds, yet studies of other taxa are needed to expand our broader understanding of the topic. While many studies have examined the nest-building behaviour of ants [54–56], O'Fallon *et al*. [57] use phylogenetically controlled comparative analyses to examine the evolution of the subterranean nests of ants. Foraging strategy is the most important variable influencing the design of nest architecture across ant species, while phylogeny plays only a negligible role, thus indicating the importance of the role of ecology in determining the structure of the subterranean nests of ants [57]. The studies published in this special issue therefore help to expand our understanding of the nests of birds and ants, thereby helping to expand the taxonomic breadth of our understanding of the evolution of nest characteristics in diverse taxa.

## 4. Large communal nests in harsh environments

Several species of eusocial insects build large communal nests in harsh environments. Perhaps most common are the termite mounds that are found in arid regions of Africa and Australia, while other termite species are responsible for the seemingly bizarre, grassless rings found in South African deserts. Such enormous structures are impressive feats of engineering, not least because of their size in relation to that of the animals constructing them [58–61].

‘Fairy circles' are conspicuous features of arid regions of the southwest coast of South Africa that consist of perennial vegetation growing within an otherwise barren and arid environment. The creation of such features was, for a long time, unclear but Juergens [62] showed that sand termites (*Psammotermes allocerus*) created such features. The termites removed vegetation following (occasional) rainfall, which subsequently collected moisture and water, which in turn sustained the growth of the vegetation at the edge of the circles, thus allowing for the long-term persistence of the termite colonies. Juergens *et al*. [63] now further show that the largest termite nests are located underneath the bare patches within the fairy circles, whilst smaller nests are located outside of fairy circles and thus have direct access to grass tussocks as food [63].

The creation of such large communal structures has invoked assertions relating to the termites being ecosystem engineers [64]. However, McAuliffe [65] suggests that such assertions may be misplaced because the earthen mounds of western South Africa, otherwise known as ‘heuweltjies' and inhabited by colonies of the western harvester termites (*Microhodotermes viator*), were traditionally built by the termites, yet they are actually the result of vegetation acting as a wind break and thus catching airborne sediment. This means that the termites do not build the mounds and so they cannot be viewed as an extended phenotype of the termites [65].

The large mounds that house termites inhabit serve to protect them against predators and desiccation, while also generating thermally stable internal climates via trade-offs between gaseous exchange, humidity and temperatures [58]. Eusocial Macrotermitinae evolved fungus-growing as a means of providing food for the termites, yet increasing ambient temperatures with ongoing climate change may mean that fungus-growing is unviable. Seymour *et al*. [66] show that the distribution of six African *Macrotermes* species is correlated with similar environmental variables; however, while three of the six species will likely lose substantial suitable habitat in a changing climate, two species will lose only a small amount of habitat and one species is predicted to gain habitat [66].

These structures are likely to be particularly important to species in harsh, arid and desert, environments in which such large communal nests are found [67]. In turn, it may be expected that such large communal nests may become particularly important to a range of species inhabiting harsh and arid environments, as part of a much wider effect of climate change on nests.

## 5. Nests in the Anthropocene

Nest-building animals face a range of issues in the Anthropocene, including the effects of increasing temperatures that result in sea-level rises that inundate nests, skewed offspring sex ratios in species with temperature-dependent sex determination, and the negative effects of including anthropogenic materials into their nests [68–70]. It is well established that seabirds [71,72] and terrestrial passerines [73,74] incorporate a range of anthropogenic materials, such as plastic fishing nets, cigarette butts and food wrapping into their nests, and that such anthropogenic material sometimes causes birds harm [71,75].

Jagiello *et al*. [76] use the outputs of a systematic literature search to compile a database regarding the occurrence of anthropogenic nest materials in birds' nests worldwide and employ phylogenetically controlled interspecific analyses to examine the main drivers of the presence of anthropogenic nest material in nests. The type of nest and the degree of sexual dimorphism between pairs of birds significantly influence the use of anthropogenic materials by birds, although the pattern of anthropogenic material use does not exhibit a phylogenetic signal, which suggests that the inclusion of such materials into nests is widespread in the world's bird species. This study thus acts as a comparative guide as to which species likely incorporate human-made components into their nests [76].

In addition to the direct effects of building nests in the Anthropocene highlighted above, increases in temperature, rainfall and wind may also indirectly influence species with temperature-dependent sex ratios. This is because the sex of offspring is dependent upon the temperature they experience during development, with one sex produced at temperatures below a threshold value and the other sex produced at temperatures above that threshold. Increasing ambient temperatures therefore skews offspring sex ratios [77], and as reptile clutches contain a disproportionate number of female offspring at higher temperatures, their populations may become unviable with further temperature increases [78,79]. Changes in the location and depth of reptile nests therefore provide key mechanisms by which they can alter nest microclimates.

Bodensteiner *et al*. [80] examine the nesting behaviour of six populations of painted turtles (*Chrysemys picta*) breeding along a broad latitudinal range and test whether changes in the selection of nest sites vary with latitudinal shifts in climate. Female turtles non-randomly selected nest sites with less canopy cover, which meant more direct sunlight and higher nest temperatures. Although there is also between-population variation in the selection of nest sites, there is no relationship with latitude or climate. This suggests that the selection of nest sites by the turtles is homogenizing nesting environments, which serves to buffer embryos from thermally induced selection. It further suggests that while the selection of nest sites is likely effective in helping turtles create optimal nest microclimates at large geographical scales, they are unlikely to enable them to adapt to rapid increases in local temperatures [80].

Changes in nest sites may not enable painted turtles to adapt to further increases in temperature [80], yet Du *et al*. [81] review the extent to which nest design allows reptiles to adapt to climate change. Du *et al*. [81] outline that reproducing females can manipulate the phenotypic attributes of offspring by selecting nest sites that increase the viability of embryos by varying nest depth, soil moisture, mean temperature and temperature variance. Despite these findings, Du *et al*. [81] caution that more studies are required to fully understand the extent to which changes in nesting behaviours can enable reptiles to adapt to climate change.

Chung *et al*. [82] assess whether plasticity in nest building behaviours enables fish to adapt to build nests in water that experiences increasingly unpredictable flow regimes in a changing climate. Wild male three-spined sticklebacks from both river and lake populations were transferred to the laboratory and allowed to build nests under static and flowing water conditions. Irrespective of provenance, males building nests under flowing water conditions take longer to construct their nests, invest more in nesting behaviour and yet build nests that contain less material, are smaller, more compact, neater and more elongated when compared to males that built nests in static water conditions. In conclusion, males show plasticity in nest-building behaviours that enables them to adapt to changes in water flow regimes [82]. The papers in this theme therefore show that nest building behaviours are influenced by changing environmental conditions, although there is evidence that reptiles and fish can adapt to climate change via changes in the location and design of their nests.

## 6. Future research directions

The studies in this special issue advance our understanding of the evolutionary ecology of nests and nesting behaviours, yet there is a need to guide the direction of further efforts in what is a rapidly moving area of research [7,9]. Each of the contributions outline those areas of research that most warrant further attention: in addition to providing an up-to-date synthesis of their specific topic, the papers in this special issue also provide the catalyst for a broad range of further studies. In particular, it is likely that novel techniques such as the modelling of nest structure [15] and artificial Intelligence techniques [16] will advance our understanding of the evolutionary ecology of nests, thus increasing interest in applications such as nest-inspired architecture [17].

Meanwhile, in the final paper of this special issue, Healy *et al*. [83] outline one of the most promising avenues for further research. It is suggested that future research should use phylogenetically controlled analyses to determine the most important evolutionary determinants of the wide variety of nest designs seen in the animal kingdom, with such studies using museum specimens of nests as a valuable, yet currently under-appreciated resource. Meanwhile, behavioural analyses of nest-building actions are much needed to better understand the cognitive ecology of nest building by birds in a social context. Such studies will help to advance our understanding of the way in which birds build the optimal nests for them.

The papers in this special issue will therefore suggest an array of further studies that will advance our understanding of the evolutionary ecology of nests. In particular, though, we propose three areas that warrant further attention. First, we intend for the papers in this issue to inspire research focusing on broad conceptual themes that go beyond individual taxa. Second, we invite further studies that will make increasing use of datasets of thousands of species to understand the evolution of nests from a broad taxonomic perspective, while empirical studies should use technology to advance our understanding as well. Third, we hope that future studies will examine how climatic and other anthropogenic changes influence nests and nesting behaviours because nests represent key ways in which animals may adapt to ongoing global change, representing a crucial current area of research.

## Data Availability

This article contains no additional data.
